# Supplementary figures and images for: Tissue-resident macrophage and dendritic cells drive type I IFN immunity to enteroviruses in the liver
Source: PLoS Pathog. 2026 Jan 27;22(1):e1013891. doi: 10.1371/journal.ppat.1013891 (PMC12858076; doi:10.1371/journal.ppat.1013891)

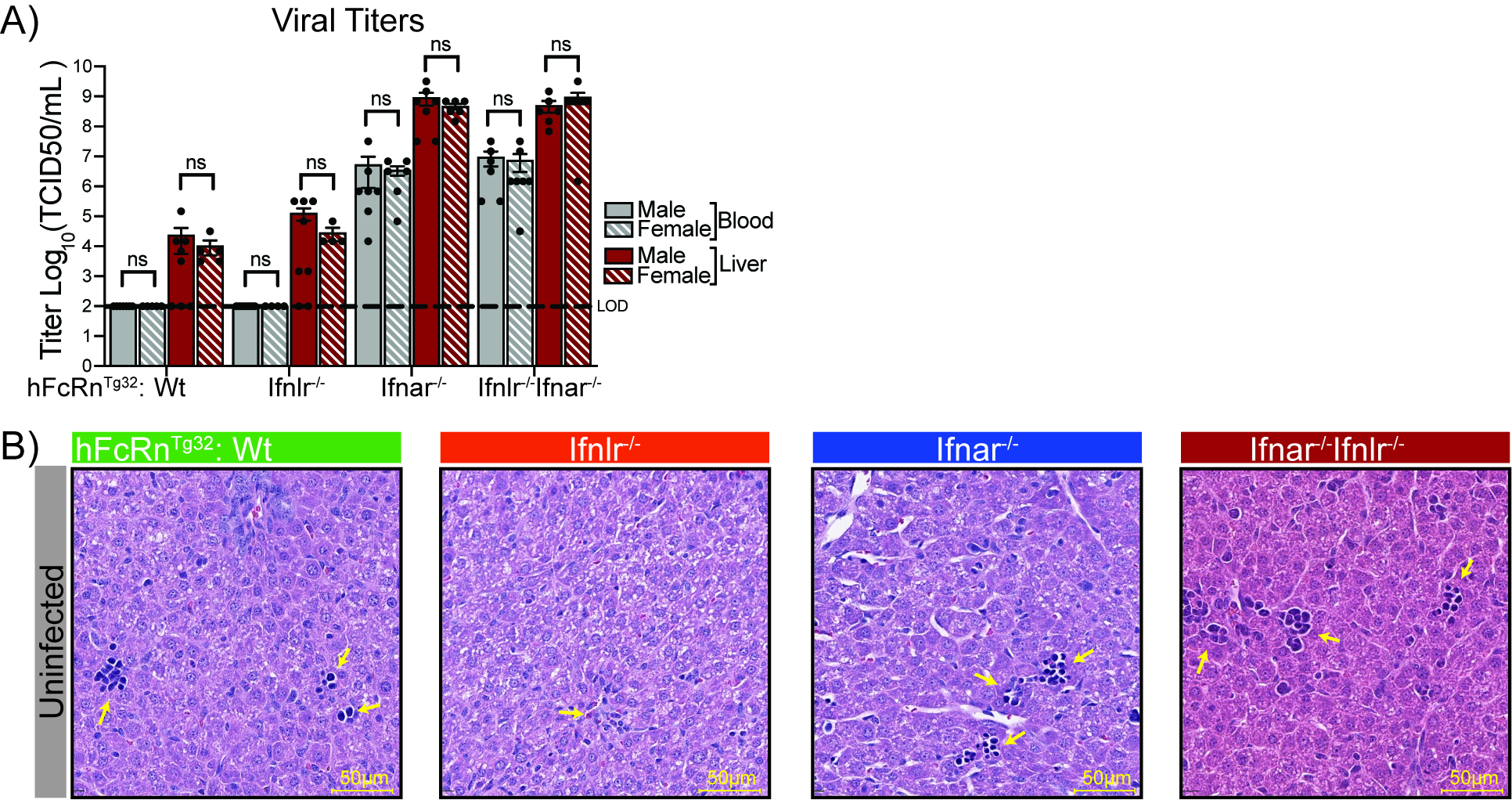

Supplement: S1 Fig — A) Viral titers of infected neonatal mice split by genotype and sex. Striped bars indicate female mice and solid bars represent male mice. Grey bars reflect blood samples and red bars reflect liver samples. Limit of detection for the assay is marked by a dashed black line. There was no significant difference (as determined by one-way ANOVA tests) between titers taken from male or female mice within each genotype across blood and liver. B) H&E images (scale bar, 50mm) of uninfected, age-matched, livers across all four genotypes. Infiltrating immune cells are indicated by yellow arrows. (TIF) [file ppat.1013891.s001.tif]

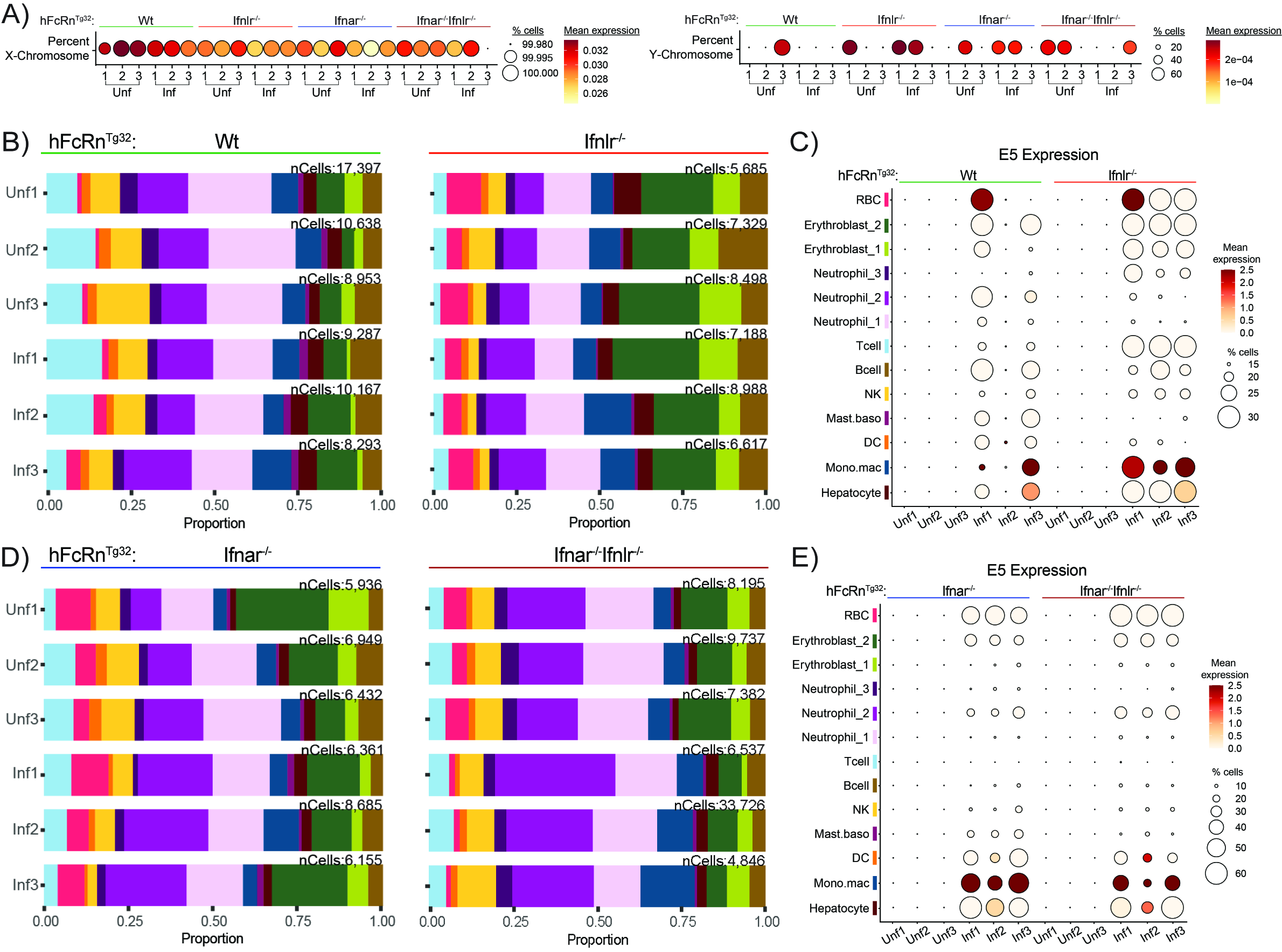

Supplement: S2 Fig — A) Dot plots visulaizing expression of X and Y linked genes for each mouse, groupe by genotype (top) and experiemntal condition (bottom). Female and male mice were equally represented across the dataset. B) Cellular composition bar plots for each mouse (left) representing Wt and Ifnlr1-/- genotypes (top), inclding the number of cells sequenced, nCells (right). C) Dot plot of scaled E5 transcript in the liver across Wt and Ifnlr1-/- genotypes split by individual mouse. Mean expression and percent of cells in which E5 transcript is found is represented by color and dot size. D) Cellular composition Bar plots and number of cells sequenced (nCells) split by individual mouse for Ifnar1-/- and Ifnar1-/- Ifnlr1-/- genotypes. E) Dot plot of scaled E5 transcript in the liver across Ifnar1-/- and Ifnar1-/- Ifnlr1-/- genotypes split by individual mouse Mean expression and percent of cells in which E5 transcript is found is represented by color and dot size. (TIF) [file ppat.1013891.s002.tif]

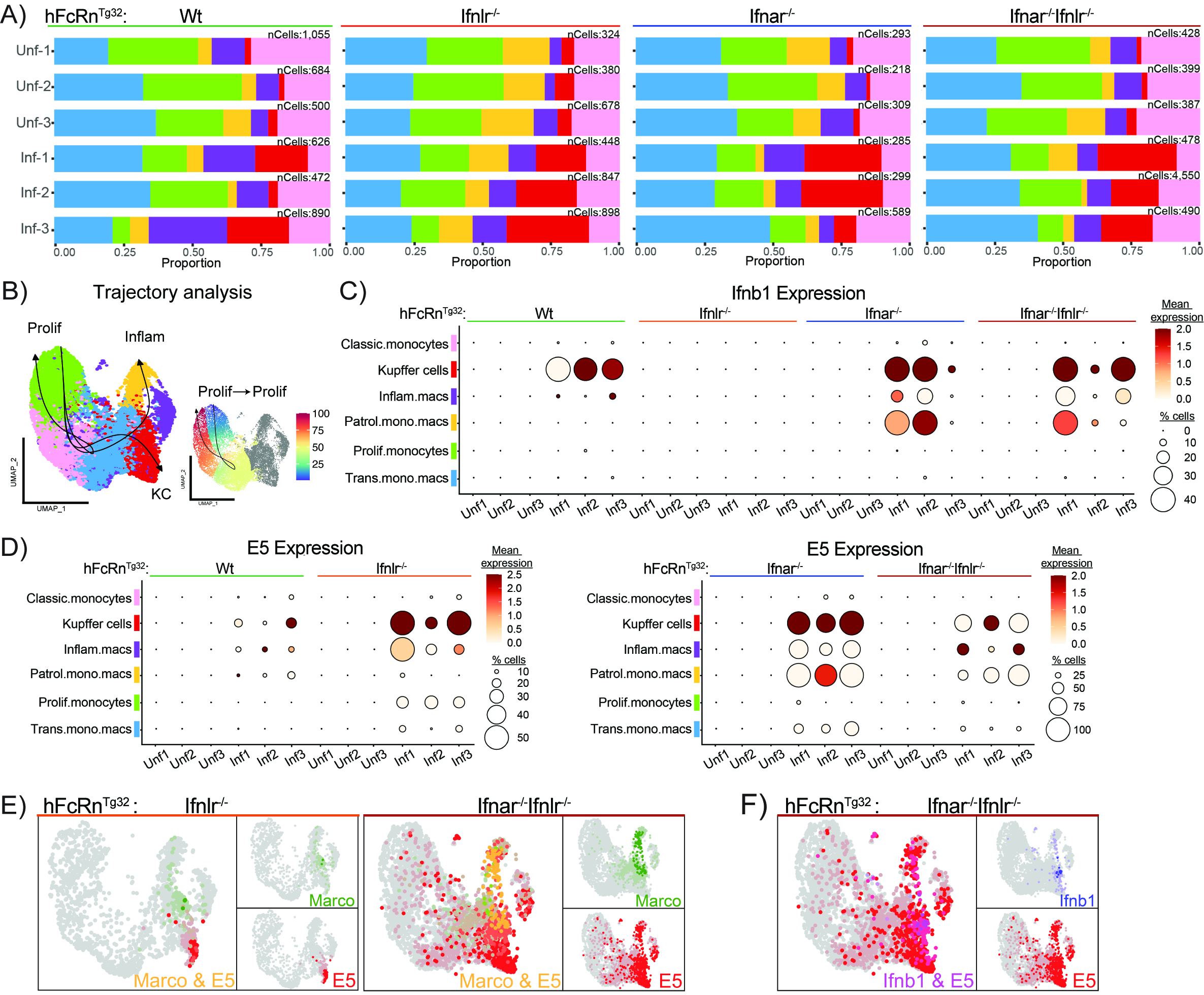

Supplement: S3 Fig — A) Bar plots depicting the proportions each cluster contributes to the overall composition of the condition split by mouse (left) and genotype (top) with nCells noted for each sample (right). B) Slingshot trajectories superimposed and labeled (Prolif, Inflam, or KC) on the reclustered Mono.mac UMAP (left panel). Feature plot of the pseutotime values (0–100) assigned to each cell along the proliferation trajectory (right panel). Color gradient (blue to red) and arrow indicate the directionality of cell differentiation along this pathway. C) Dot plot of Ifnb1 induction by cell type (y-axis) split by genotype (top) and infection status (x-axis) for all mice present in the dataset. Expression data was scaled to allow for comparison of Ifnb1 response across genotypes with color reflecting mean gene expression and dot size reflecting proportion of the cells in each cluster that contain the gene of interest. D) Dot plots of scaled mean expression of E5 split by cell type (y-axis), genotype (top), and mouse (x-axis). Left panel reflects Wt and Ifnlr1-/- average proportion of E5 containing cells for each cluster (between 0–50%). In contrast, the right panel reflects Ifnar1-/- and Ifnar1-/- Ifnlr1-/- genotypes whose average proportion of E5 containing cells for each cluster is between 25–100%. E) Merged Feature plots looking at expression of key KC marker, Marco (green) and E5 (red) across infected Ifnlr1-/- and Ifnar1-/- Ifnlr1-/- samples. Cells expressing both genes appear as yellow dots. F) Merged Feature plot showing expression of Ifnb1 (blue) and E5 (red) in infected Ifnar1-/- Ifnlr1-/- samples. Cells expressing both genes appear as purple dots. The Ifnlr1-/- samples did not induce detectable Ifnb1 in response to infection, therefore, a complimentary Feature plot could not be produced for this genotype. (TIF) [file ppat.1013891.s003.tif]

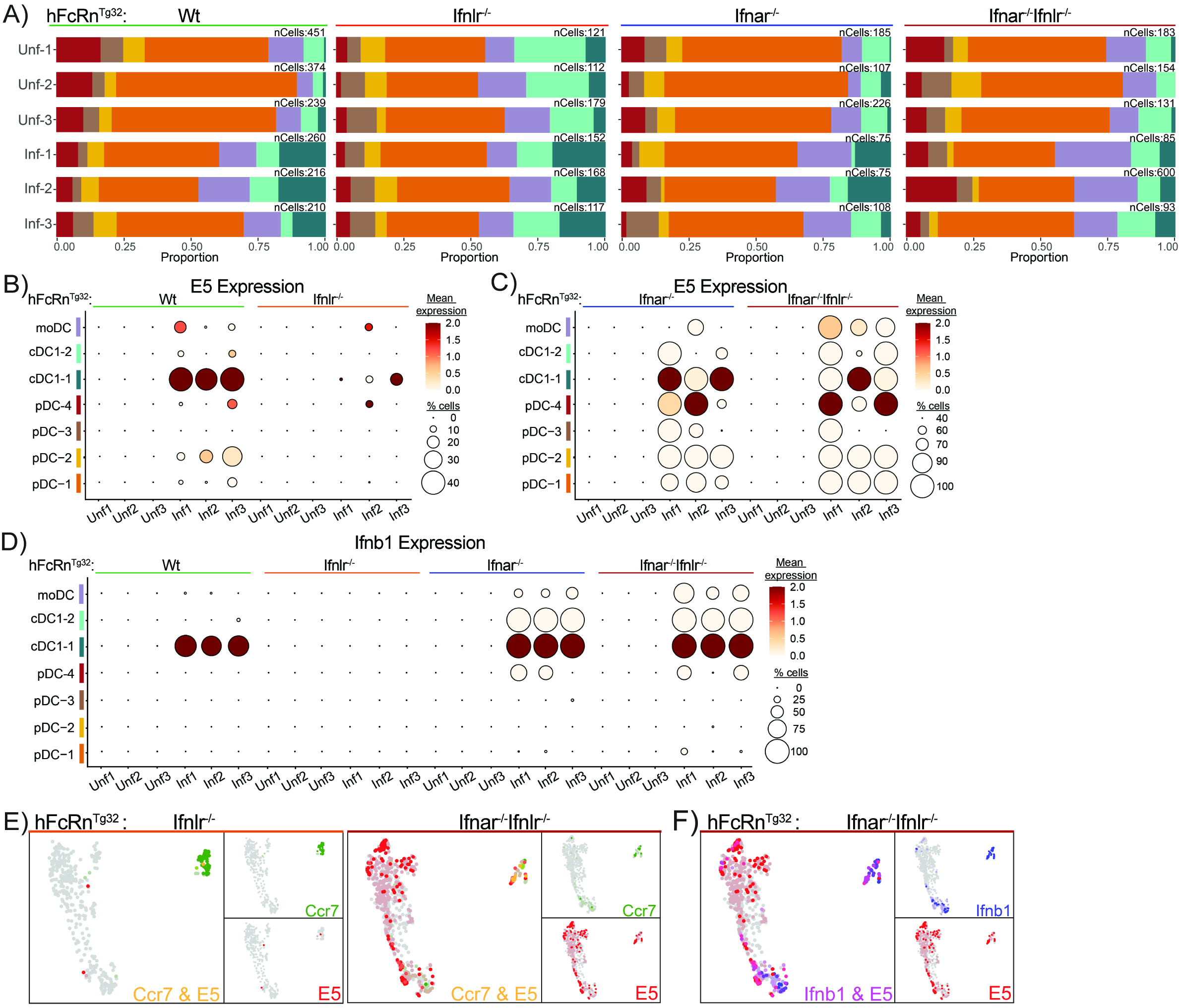

Supplement: S4 Fig — A) Bar plots depicting the proportions each cluster contributes to the overall composition of the experimental condition. Plots are split by genotype (top), individual mouse (left), and proportion (bottom), with nCells annotated to the right of each sample. B) Dot plot of Ifnb1 production by cell type, split by genotype and infection status for all mice present in the dataset. Expression data was scaled to allow for comparison of Ifnb1 response across genotypes with larger circles representing a greater proportion of the cells in that cluster having reads and darker red indicating clusters with higher gene expression. C) Dot plots of scaled mean expression of echovirus split by cell type (y-axis), genotype (top), and mouse (x-axis) for Wt and Ifnlr1-/- samples. D) Dot plots of scaled mean expression of echovirus split by cell type (y-axis), genotype (top), and mouse (x-axis) for Ifnar1-/- and Ifnar1-/- Ifnlr1-/- samples. E) Merged Feature plots looking at co-expression of key dendritic cell marker, Ccr7 in green, and E5 in red across infected Ifnlr1-/- (left) and Ifnar1-/- Ifnlr1-/- (right) samples. Cells expressing both genes appear as yellow dots. F) Merged Feature plot showing co-expression of key Ifnb1 in blue and E5 in red in infected Ifnar1-/- Ifnlr1-/- samples. Cells expressing both genes appear as purple dots. Ifnlr1-/- samples did not induce detectable Ifnb1 in response to infection, therefore, a complimentary Feature plot could not be generated for this genotype. (TIF) [file ppat.1013891.s004.tif]

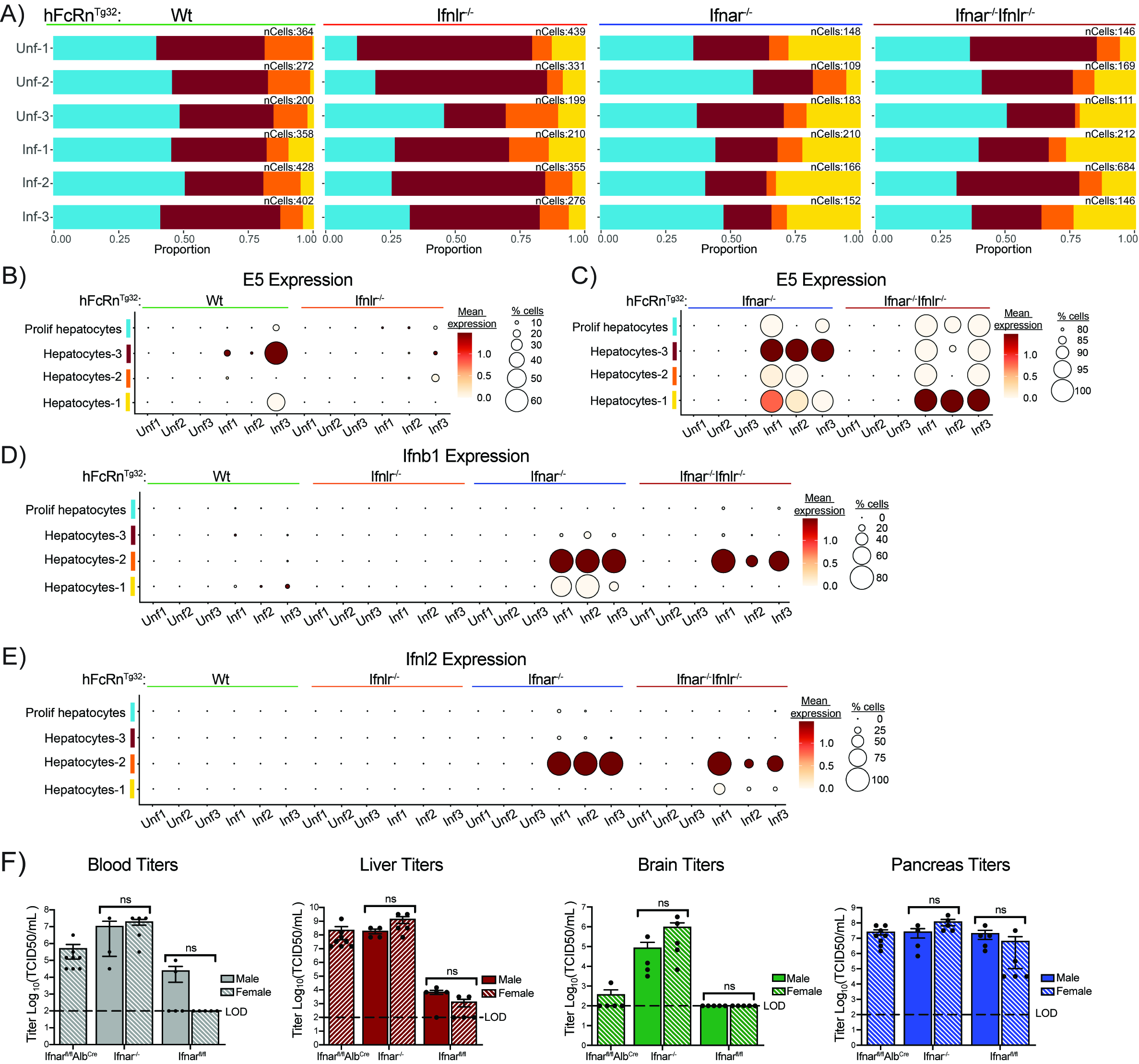

Supplement: S5 Fig — A) Cellular proportion bar plots and number of cells sequenced (nCells) split by individual mouse for all genotypes. Annotations include genotype (top), infection status (left), total proportion (bottom), and nCells (right). B) Dot plot showing the average expression and proportion of cells expressing echovirus-5 across the different hepatocyte clusters (y-axis) separated by genotype (top) and individual mouse (x-axis). C) Dot plot of scaled E5 transcript in hepatocytes across Wt and Ifnlr1-/- genotypes split by individual mouse (x-axis) and grouped by genotype (top). D) Dot plot showing scaled Ifnb1 transcript across hepatocytes clusters and genotypes split by individual mouse (x-axis) and grouped by genotype (top). E) Dot plot of scaled Ifnl2 transcript across hepatocytes clusters and genotypes split by individual mouse (x-axis) and grouped by genotype (top). F) Echovirus titers from key secondary sites of infection including the blood, liver brain, and pancrease. Titers were determined by TCID50 on organs harvested from all three genotypes (hFcRnTg32: Ifnar1-/-, Ifnar1fl/fl, Ifnar1fl/fl AlbCre), split by sex to confirm the lack of sex-bias during infection. Limit of detection for the assay is marked by a dashed black line. There was no significant difference (as determined by one-way ANOVA tests) between titers taken from male and female mice within each genotype across all four organs. (TIF) [file ppat.1013891.s005.tif]

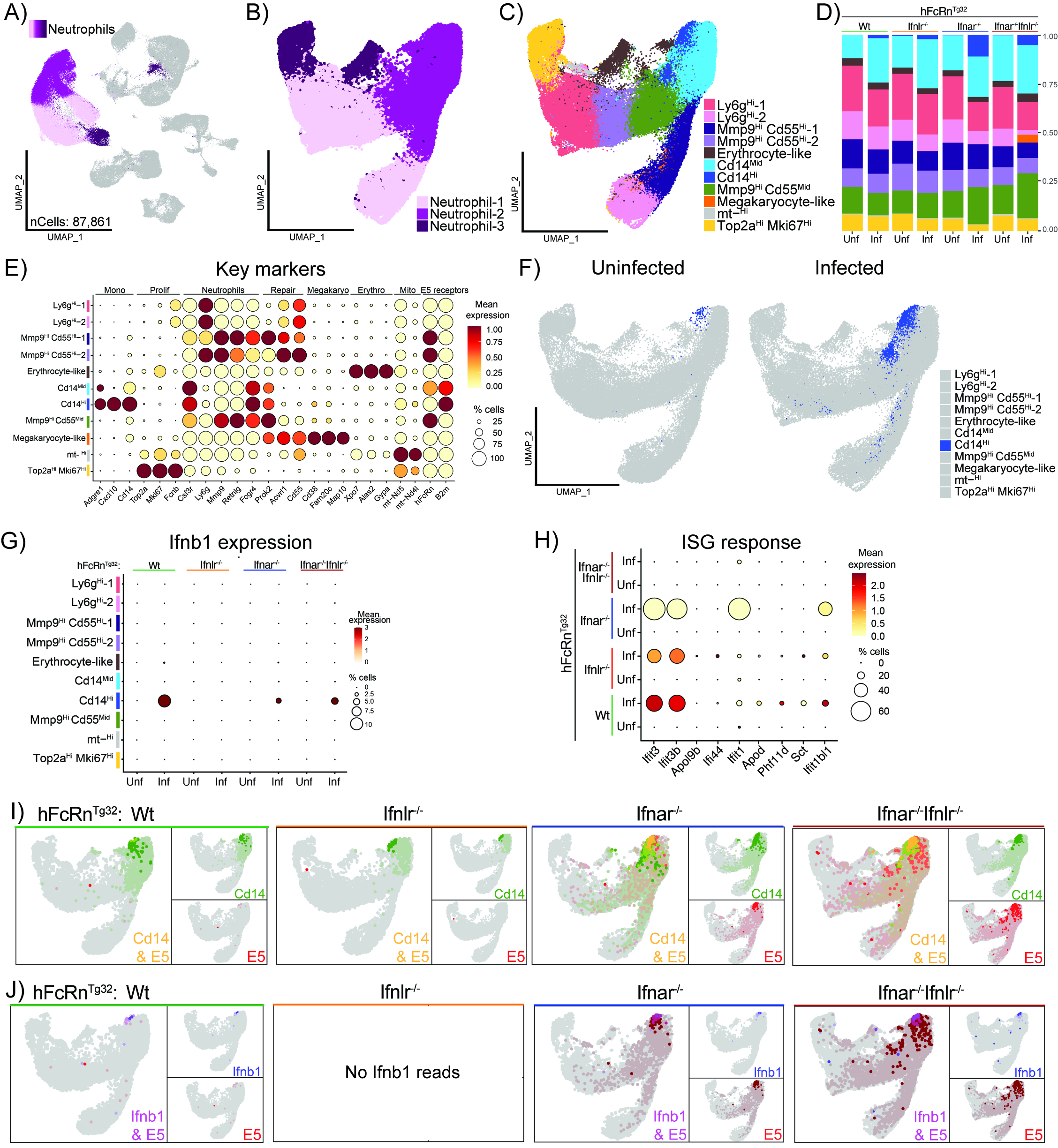

Supplement: S6 Fig — A) UMAP highlighting the three neutrophil clusters in the original whole-liver dataset. These populations consist of 87,861 cells across all 8 experimental conditions. B) UMAP of the reclustered neutrophil population with the original identities assigned: Neutrophil-1 (light pink), Neutrophil-2 (magenta), Neutrophil-3 (purple). C) UMAP of the reclustered neutrophil population with new cell identities were assigned based on key marker expression. Cell types are annotated as follows: Ly6gHi (-1, dark pink, and -2, light pink), Mmp9HiCd55Hi (-1, dark purple and -2, light purple), Erythrocyte-like (brown), Cd14Mid (light blue), Cd14Hi (dark blue), Mmp9HiCd55Mid (Green), Megakaryocyte-like (orange), mitochondrial-high (mtHi, grey), and Top2aHiMki67Hi (yellow). D) Bar plot showing the proportion of each cell cluster within the subsetted dataset, split by genotype (top) and infection status (bottom). E) Key markers (genes on x-axis) for each cell type (y-axis) including E5 receptors are shown as a Dot plot. Scaled mean expression is shown as a color scale and percent of cell expressing each gene is reflected by the size of the dot. Labels on top of the graph indicate gross groupings of key genes. F) UMAP of the reclustered dendritic cell population split by infection status to highlight the difference in abundance of the Cd14Hi cluster (dark blue). G) Dot plot showing the average expression and proportion of cells expressing Ifnb1 across the different cell types (y-axis) separated by genotype (top) and infection status (x-axis). H) Dot plot showing the average expression and proportion of cells expressing panel of genes used for the ISG-score across genotype and infection status. I) Merged Feature plots of infected Wt (left) and Ifnar1-/- (right) samples showing the co-expression of a key Cd14Hi marker Cd14 (green) and E5 (red). Yellow indicates that a given cell expresses both Cd14 and E5. J) Merged Feature plots of infected Wt (left) and Ifnar1-/- (right) samples showing [file ppat.1013891.s006.tif]
