# Supplementary material for: Tissue-resident macrophage and dendritic cells drive type I IFN immunity to enteroviruses in the liver
Source: PLoS Pathog. 2026 Jan 27;22(1):e1013891. doi: 10.1371/journal.ppat.1013891 (PMC12858076; doi:10.1371/journal.ppat.1013891)
Supplement: Attachment — Submitted filename: Rebuttal Letter_emh.docx [file ppat.1013891.s019.docx]

**Reviewer #1**

…Reliant solely on scRNA seq data without any further validation or measurement of protein levels. Please quantify the interferon protein levels in the different genotypes, at least in the bulk liver lysates.

We appreciate the reviewer’s comment regarding validation of the scRNA-seq findings at the protein level. To address this, we performed mouse Luminex protein quantification of key antiviral interferons, including IFNα, IFNβ, and IFNλ2, using the same whole-liver lysates analyzed for viral quantification in Figure 1, panel B. These results demonstrate that all four genotypes induced both type I and type III IFNs during echovirus infection, with the Ifnar-deficient genotypes showing higher protein levels at 40 hpi. These new data have been incorporated into the revised manuscript as **Figure 1, panel E** and are addressed in the Results section of the revised manuscript at **Lines 156–164**.

Limited translation potential, as the value of the mouse data to human infections is unclear. Study limitations, including translational relevance and model system constraints, are not sufficiently addressed in the manuscript text.

We agree that emphasizing the translational boundaries of mouse models is important. In response, we have expanded the discussion to more clearly outline the limitations of extrapolating murine data to human infections. The revised text acknowledges that while mouse models are indispensable for dissecting mechanistic immune responses, species-specific differences in immune regulation and viral receptor usage necessitate cautious interpretation. These points are now discussed in detail. Addressed in the discussion section of the revised manuscript at **Lines 652–665.**

A single Echovirus serotype, Echovirus 5, and a single strain (Noyce) of this serotype are used to generalise findings for echovirus infections. Noyce strain has been shown to interact with cell surface heparan sulfate, which can be different between mice and humans.

We have added text clarifying that Echovirus 5 (Noyce strain) serves as a representative model for the echoviruses, consistent with prior work from our laboratory. As hFcRn functions as a pan-echovirus receptor, we believe that our findings remain broadly applicable to other echovirus serotypes. This is addressed in the results section of the revised manuscript at **Lines 134–135**.

Use of adult mice for conditional hepatocyte KO is a technical necessity, but this is not discussed and assumed to be similar in the neonatal setting.

We have cited the foundational paper describing age-related excision of Albumin in these conditional mouse models and noted that prior work in adult mice also demonstrated the critical role of type I IFNs in restricting echovirus infection. Addressed in the discussion section of the revised manuscript at **Lines 665–672.**

The viral titres used for the genotypes are significantly different, and while the reasons are clear, it can have an impact on the cellular response. This needs to be discussed further.

To clarify, this study was designed to capture the innate immune landscape during early acute infection (40 hpi) across various IFN knockout models. The viral doses were optimized for each genotype to enable meaningful comparison of infection dynamics while avoiding overwhelming infection in highly susceptible strains. This is addressed in the results section of the revised manuscript at **Lines 140–143.**

Immunofluorescence for localisation of specific proteins, as well as dsRNA or viral protein, with cell markers, is necessary to support the conclusions.

We agree with the reviewer that validating localization of viral RNA and host markers strengthens the study’s conclusions. Accordingly, we have added RNAScope images of uninfected and infected WT livers, assessing co-expression of the canonical Kupffer cell marker (Adgre1) and viral RNA. These findings are now included in **Figure 4, panels J and K**. Addressed in the results section of the revised manuscript at **Lines 387–393**.

The data presented in Figure 3 is based on the intersection between two gene sets, viz., the wild type vs type 1 deficient. Based on this, 17 shared ISGs are identified. However, without a similar analysis for type 3, the role of type 3 during infection cannot be precluded. Please perform this analysis.

In response, we have revised our gene selection approach to ensure a more comprehensive analysis. **Panels D–N of Figure 3** have been updated to reflect this expanded ISG list, and a new **Supplemental Table 3** has been added, detailing all genes incorporated into the updated analysis. This is addressed in the results section of the revised manuscript at **Lines 284–291.**

**Minor Revisions:**

Line 58, it is stated that fatality rises to 78%. Please specify that this is the case fatality ratio.

We have clarified that this value represents the *case fatality rate*. Addressed in the introduction section of the revised manuscript at **Line 75**.

In line 119, it is stated that wt and ifnlr1- mice survived beyond this early acute time point and refers to figure 1A. How long was this monitored, and what was the percentage survival?

All mice from these two genotypes survived past the 7dpi observation window, after this point they we’re euthanized as determined by the study design. This information has now been included in the Materials and Methods section to enhance transparency. This is addressed in revised manuscript at **Lines 752–755**.

In line 156, 40 hpi is chosen for scRNA seq. What is this based on? Please provide a rationale for this choice in this section.

We have clarified the rationale for selecting 40 hpi, emphasizing that this time point captures peak innate immune activation prior to extensive tissue pathology. This is addressed in the results section of the revised manuscript at **Lines 141–143**.

In lines 169–176, markers for the various cells are listed. Where do these markers come from? The dataset or libraries used for the clustering are also not clear. Please add this to the methods.

The markers used for cell-type identification were derived from established, peer-reviewed literature rather than external libraries, as current reference libraries insufficiently capture the heterogeneity of neonatal liver cell populations. We have added the citations used to assign cell types to the revised manuscript at **Line 205**.

Line 359 says cDC cooperation with Kupffer cells. The data presented is not sufficient to claim this and only shows that cDCs also have a type 1 response. Please adjust.

We agree that this section required refinement. The title and narrative have been adjusted to more accurately reflect the observed data, focusing on concurrent activation rather than functional cooperation. Addressed in revised manuscript at **Line 425**.

Line 548 of the discussion states that it remains possible that KCs are not productively infected. Yet, the results section is presented as if they are. This needs to be stated earlier, and the results adjusted accordingly. As these cells are phagocytic, this cannot be dismissed without evidence.

We have revised the results section to explicitly state that, while our data confirm productive infection at the organ level, the extent of infection within specific cell types remains undetermined. Addressed in revised manuscript at **Lines 225–230**.

Based on the scRNA seq analysis presented from line 671 onwards, it appears that doublets and multiplets have not been removed. Please explain why.

Our data processing pipeline includes explicit thresholds for nCells and nFeatures that effectively remove doublets and multiplets. We have clarified this in the methods section. Addressed in revised manuscript at **Lines 818–823.**

The QC for the scRNA seq is not described sufficiently, and the QC data are not presented. Please add this.

We now include a high-level description of QC methods within the text, along with a link to the publicly available, figure-by-figure code and raw data repository. Addressed in revised manuscript at Lines **807–838.**

It is highly recommended that the ARRIVE guidelines be followed for the reporting of the in vivo experiments.

The manuscript has been reviewed for compliance with ARRIVE 2.0 guidelines, and corresponding details have been added to the Materials and Methods section. Addressed in revised manuscript at **Lines 735–757.**

Ethical statements for the animal work are missing.

We have added a comprehensive ethical statement to the Materials and Methods section. Addressed in revised manuscript at **Lines 694–697**.

In addition to these, the broad conclusions of the mouse work on human infection must be tempered. Furthermore, the limitations of the study must be discussed in detail. Please include the following: Highlight that this is work in mice, and the translation to humans remains to be seen. Please refrain from presenting the mice data as conclusive evidence of human disease, as this is how the work currently reads. Please discuss the differences between the human and mouse liver.

We fully agree with the reviewer that appropriate contextualization is critical. The discussion has been expanded to emphasize that these findings are derived from mouse models and that translational extrapolation to human infection remains speculative. The section now also includes discussion of key differences between human and murine liver immune composition. Addressed in revised manuscript at **Lines 652–665.**

Please discuss the differences in infection dosages and the consequences. For instance, the wt and type 1 deficient mice have a higher viral load, which could result in earlier detection and better response.

As our study focused on capturing innate responses at early acute stages, viral challenge doses were adjusted to ensure measurable infection while avoiding lethality in highly susceptible genotypes.

The conditional KO is performed in adult mice and so cannot be extrapolated to what would happen in neonatal mice. Considering this is the primary motivation for the manuscript, this limitation must be discussed.

As mentioned, it was essential to perform these experiments in adult animals, as the conditional *Albumin* knockout model is only effective in adults and does not function in the neonatal context. Consequently, the conditional KO experiments cannot be directly extrapolated to neonatal physiology, which is the primary focus of our study. However, we have previously demonstrated that type I interferons restrict echovirus infection in adult mice (PMID: [33513208](https://pubmed.ncbi.nlm.nih.gov/33513208/)), suggesting that similar antiviral pathways are likely operative in the neonatal and adult settings. We have now clarified this rationale and explicitly discussed this limitation in the revised Discussion, **Lines 665–672**.

**Reviewer #2**

The data confirm the indispensable role of IFN-I; however, the conclusions regarding its cellular sources rely solely on scRNA-seq, without complementary experimental validation. The contribution of Kupffer cells and cDC1s to IFN-I–mediated restriction was not directly tested. Monocytic macrophages and DCs also expressed ISGs (Fig. 2), suggesting additional roles. Depletion experiments targeting Kupffer cells or cDCs in vivo, or CD11c-cre/Clec4f-cre × Ifnar flox models, would clarify their specific contributions in IFN production and responses.

We fully agree that defining the cellular sources of IFN-I *in vivo* would be an important extension of this work, and we argue that our scRNA-seq data provide strong evidence supporting our conclusions regarding Kupffer cell and cDC1 IFN induction. However, deeper animal model–based experimentation falls beyond the scope of the current study, which focuses specifically on how type I IFN signaling to hepatocytes influences infection outcomes in neonatal models. Generating and backcrossing the multiple Cre × Ifnar^flox^ lines required for such experiments, while also incorporating hFcRn expression and mFcRn knockout to permit echovirus infection, would require extensive time. We have clarified this limitation and noted it as an important direction for future investigation in the **Discussion (Lines 606–611).**

In addition, viral RNA in scRNA-seq is used as the only evidence of infection. As the authors acknowledge, viral RNA in phagocytes could reflect uptake of infected material. Confirmation by staining viral proteins/RNAs in situ (as in Fig. 1D) with co-localization of the proposed infected cells via confocal microscopy or flow cytometry would strengthen this conclusion.

To address this, we performed RNAScope analysis on uninfected and infected WT livers, probing for co-expression of viral RNA and the canonical Kupffer cell marker *Adgre1*. These data demonstrate the presence of viral RNA within liver macrophages and are now included as **Figure 4, panels J and K**. Addressed in the results section of the revised manuscript at **Lines 387–393.**

Fig. 1D shows more disseminated viral RNA in Ifnar/Ifnlr double KO mice compared with Ifnar KO, but this is not reflected in TCID50 data (Fig. 1B). Quantification of imaging would help clarify a potential role of IFN-III in the absence of IFN-I.

We have now included HCR images spanning larger regions of the liver to highlight the regional heterogeneity of echovirus infection. These tile scans are complemented by higher-magnification images illustrating differences in viral abundance between resistant (WT and Ifnlr-/-) and highly susceptible (Ifnar-/- and Ifnar-/-Ifnlr-/-) genotypes. These data are incorporated as **Figure 1, panel D**. Addressed in revised manuscript at **Lines 153–155**.

**Minor Revisions:**

E5 expression in scRNA-seq was mostly relegated to Supplementary Materials. Since it is central to correlating infection with IFN-I expression, it should be shown in the main figures.

We have ensured that the echovirus transcript data are clearly represented in the main figures. Specifically, **Figure 2D–G** highlight viral RNA distribution across genotypes and cell types, shown both as UMAP and Dot plots.

Fig. 2B: Abbreviation definitions are unclear, possibly due to formatting.

We have reformatted the figure to ensure all abbreviations are clearly defined and aligned.

Fig. 2C: Expression of B2m and hFcRn appears higher in innate immune cells than hepatocytes. As viral RNAs localized mainly to macrophages in resistant strains (WT, Ifnlr KO) but to hepatocytes in IFN-I–deficient strains, validation of hFcRn expression by IF or flow cytometry would be valuable.

FcRn is well documented to exhibit higher expression in immune cells, where it plays an essential role in IgG and Albumin trafficking. The variation in hFcRn expression across cell types observed in our dataset is consistent with its known physiological roles. We therefore interpret our results as reflecting differences in antiviral signaling rather than receptor expression alone [1, 2]

L255–256: Cxcl10 expression was highest in Ifnar/Ifnlr double KO mice. This requires explanation.

The original analysis that included *Cxcl10* has been revised, as we updated the ISG scoring gene list. *Cxcl10* is no longer part of this revised analysis. Addressed in revised manuscript at **Lines 284–291.**

L257–259: Fig. 3A–C show type I IFNs mainly from DCs in susceptible strains, while in WT only IFN-β is expressed. This does not support the conclusion that four clusters produced IFNs across all genotypes.

Our data show differential induction of type I (α and β) and type III (λ) interferons across genotypes, reflecting distinct kinetic profiles. As type I IFN induction is transient in models capable of mounting effective antiviral responses, its sustained expression in Ifnar-deficient genotypes likely represents an unsuccessful compensatory attempt to control infection. These interpretations are supported by both the Luminex data **(Figure 1E**) and scRNA-seq results (**Figure 3A–C**).

L301–311: Pseudotime analysis suggesting Kupffer cells are not derived from monocytes requires experimental validation (e.g., Ccr2 KO). If not feasible, I recommend removing this section.

We thank the reviewer for raising this concern. However, our pseudotime analysis does not claim Kupffer cells are independent of monocyte derivation. Rather, the analysis suggests that Kupffer cells and inflammatory macrophages (Inflam.macs) represent distinct yet related monocyte-derived populations.

L312: The statement that Mono.mac was the major source of IFN-I is not consistent with Fig. 3A–C and requires clarification.

Among the 13 cell clusters identified, the Mono.mac cluster is one of three that produce significant levels of type I IFNs. Thus, we consider it a major contributor to IFN-I production during infection.

L446–451: Sentence repetition.

We thank the reviewer for noticing this. The repeated sentence has been removed.

L507–509: Please clarify whether neonatal and aged mice show different immune responses in hFcRn(Tg32) background.

We have previously used the hFcRn (Tg32) mouse model to study echovirus infection and demonstrated that type I IFNs potently restrict viral replication in adult animals [3, 4]. These findings indicate that the antiviral role of IFN-I is conserved across developmental stages. While the current study focuses on neonatal infection, our prior work in adults suggests that similar innate immune mechanisms are likely operative in both settings, albeit with potential differences in magnitude and kinetics reflecting developmental context. We have clarified this point in the revised Introduction **(Lines 92–103).**

L538: Evidence for Kupffer cell expansion comes only from scRNA-seq. Experimental validation (e.g., Ki67, EdU) is needed.

We agree that direct validation of Kupffer cell proliferation would further substantiate our findings; however, such experiments present substantial technical challenges given the very low frequency of Kupffer cells in the neonatal liver and the difficulty of performing EdU or Ki67 labeling within this developmental window. One of the key strengths of single-cell transcriptomic analysis is its ability to sensitively detect rare proliferative states within low-abundance populations, as we observed here. To acknowledge this, we have clarified in the revised Discussion that the apparent Kupffer cell expansion represents a transcriptomic signature consistent with proliferation rather than definitive evidence of cell division, and we note this as an important direction for future in vivo validation (**Lines 612–625**).

**Reviewer #3**

I had several concerns regarding the interpretation of data from the Ifnar-/-Ifnlr-/- mice. In Figure 1, the authors show that viral titers are not significantly different from WT mice in the liver of the Ifnlr-/- mice. However, the level of inflammatory infiltrates was not quantified in Fig. 1C. It seems possible that differences in inflammation levels may exist between groups despite similar viral titers. It would be helpful to see pathology scoring performed for the H&E histology. Comparing the images shown for the Ifnar-/- and the Ifnar-/-Ifnlr-/- mice, there appears to be more inflammatory infiltrate in the DKO mice.

While we agree that blinded histopathologic scoring can provide supportive information, our single-cell RNA-seq data already offer a high-resolution and quantitative view of immune cell composition and activation states within the infected liver, including clear evidence of increased myeloid and inflammatory cell populations in the Ifnar-/- Ifnlr-/- mice. Furthermore, we have previously characterized liver histopathology and functional changes in mice following echovirus infection, demonstrating similar inflammatory phenotypes [3, 4]. Given the consistency of these findings and the detailed immune profiling already included here, we believe additional scoring would not substantially alter the interpretation. We have clarified this point in the Discussion to emphasize that the observed immune cell changes are supported by both transcriptomic and prior histologic evidence.

Related to the comment above, there seem to be more cells containing viral RNA in the Ifnar-/-Ifnlr-/- DKO than in the Ifnar-/- mice. Providing some quantification of the HCR data would be helpful here, or RT-qPCR data to measure viral positive and negative sense RNA in the different groups. In particular, examining negative-sense viral RNA would strengthen the analysis, as this is likely not detected in the scRNA-seq data.

To address this point, we have expanded our imaging dataset to include HCR tile scans of larger liver sections, highlighting regional infection patterns. These images, together with magnified insets, illustrate the increased viral abundance in Ifnar-/- and Ifnar-/-Ifnlr-/- mice compared with resistant genotypes. These additions have been incorporated as **Figure 1, panel D**.

**Minor Revisions:**

There is a typo on line 490 'live' should be 'liver.'

We thank the reviewer for catching this typographical error, which has been corrected in the revised manuscript.

1.	Fan YY, Avery LB, Wang M, O'Hara DM, Leung S, Neubert H. Tissue expression profile of human neonatal Fc receptor (FcRn) in Tg32 transgenic mice. MAbs. 2016;8(5):848-53. Epub 20160422. doi: 10.1080/19420862.2016.1178436. PubMed PMID: 27104806; PubMed Central PMCID: PMCPMC4968098.

2.	Latvala S, Jacobsen B, Otteneder MB, Herrmann A, Kronenberg S. Distribution of FcRn Across Species and Tissues. Journal of Histochemistry & Cytochemistry. 2017;65(6):321-33. doi: 10.1369/0022155417705095. PubMed PMID: 28402755.

3.	Wells AI, Grimes KA, Kim K, Branche E, Bakkenist CJ, DePas WH, et al. Human FcRn expression and Type I Interferon signaling control Echovirus 11 pathogenesis in mice. PLOS Pathogens. 2021;17(1):e1009252. doi: 10.1371/journal.ppat.1009252.

4.	Wells AI, Grimes KA, Coyne CB. Enterovirus Replication and Dissemination Are Differentially Controlled by Type I and III Interferons in the Gastrointestinal Tract. Mbio. 2022;13(3):e0044322. Epub 20220523. doi: 10.1128/mbio.00443-22. PubMed PMID: 35604122; PubMed Central PMCID: PMCPMC9239134.
