# Supplementary material for: Tissue-resident macrophage and dendritic cells drive type I IFN immunity to enteroviruses in the liver
Source: PLoS Pathog. 2026 Jan 27;22(1):e1013891. doi: 10.1371/journal.ppat.1013891 (PMC12858076; doi:10.1371/journal.ppat.1013891)
Supplement: Attachment — Submitted filename: Rebuttal Letter_emh_CC_v2 (1).docx [file ppat.1013891.s020.docx]

**Part II – Major Issues: Key Experiments Required for Acceptance**

Reviewer #1: (No Response)

Reviewer #2:

1. Clarification of the cellular source of IFN-I
The newly added section (lines 617–623) focuses primarily on IFN-I–responsive cells rather than the cellular sources of IFN-I, which was the central issue raised previously. Although additional mouse crosses are time-consuming, further experimental support is necessary if the manuscript is to assert mechanistic interactions between Kupffer cells and dendritic cells in driving IFN-I responses.

We agree with the reviewer that additional experimental approaches would be required to define mechanistic interactions between Kupffer cells and dendritic cells. In the present study, however, we do not intend to assert a hierarchy of activation between these populations; rather, our data support the interpretation that these cell types contribute to IFN-I production in parallel within the limitations of the current dataset. To address this concern, we now include changes to the text as follows (with edits highlighted in red) and well as modifying the title of the manuscript to “*Tissue-Resident Macrophage and Dendritic Cells Drive Type I IFN Immunity to Enteroviruses in the Liver*”, removing mention of “cooperation”.

“If sentinel cells, such as Kupffer cells, dendritic cells, or neutrophils, cannot respond to type I IFNs produced during initial infection, we propose that the host must rely on direct viral sensing to trigger downstream responses. This shift may delay the initiation of protective immunity, permitting broader viral dissemination before control mechanisms are fully engaged. Therefore, we suggest a model in which both hepatocyte susceptibility and the functional capacity of innate immune cells to initiate and respond to IFNs likely contribute to overall echovirus pathogenesis and this should be considered in future studies of enterovirus-induced organ failure.”

2. Colocalization of viral RNA in Kupffer cells
The inclusion of new imaging data is appreciated. However, the colocalization between viral RNA signals and Kupffer cell markers remains difficult to interpret with confidence. Higher-resolution confocal microscopy in single xy-plane images and quantitative colocalization analysis would significantly strengthen this conclusion.

In this second revision, we have added higher resolution images to **Figure 4 (panel L)**. This change has been reflected in the text in **lines 391-400**. We have also quantified the number of Echovirus positive cells that are also positive for the macrophage marker Adgre1 via unbiased image analysis. To achieve this, we captured (at random) ten 40x, images of Echovirus positive cells. Using this approach, we identified that ~96% of echovirus positive cells in the liver of immunocompetent mice were also positive for the macrophage marker Adgre1. This aligns with our single cell findings. Additionally, no echovirus was detected in our uninfected controls, however, Adgre1 probe sets were seen uniformly throughout the liver (**Figure 4, panel J**).

Reviewer #3: NA

**Part III – Minor Issues: Editorial and Data Presentation Modifications**

Reviewer #1: (No Response)

Reviewer #2:

1. Strength of conclusions regarding Kupffer cell–derived IFN-I and Kupffer cell expansion

While I appreciate the technical challenges associated with generating additional genetic models to directly assess IFN-I production by Kupffer cells, and likewise the difficulty of evaluating Kupffer cell expansion during echovirus infection, the current data do not provide strong support for the conclusions presented. I recommend tempering these claims, particularly the statements in lines 612–613 of the Discussion.

We appreciate the concern reviewer 2 has for potential misinterpretation of our conclusions. We have tempered the language used in lines 624-625 accordingly. We have amended the text as follows:

“A key finding of this study is the ~~robust and~~ selective expansion of Kupffer cells during echovirus infection, accompanied by their dominant role in type I IFN production.”

2. Interpretation of pseudotime analysis

In the pseudotime section (lines 353–364, R1 version), the manuscript states that Kupffer cells and inflammatory macrophages appear to be distinct but related monocyte-derived populations. Despite this, the subsequent conclusion (lines 362–364) that “the expansion of Kupffer cells during infection is not the result of infiltrating inflammatory macrophages repopulating the resident Kupffer cell niche” is stronger than the data support. As written, this conclusion is potentially misleading and should be revised to align with the evidence.

We have clarified our pseudotime analysis findings for clarity as follows: Lines 355-367

“To confirm that the expanding Kupffer cell cluster represented a distinct population from the infiltrating inflammatory macrophage cluster, we performed trajectory analysis on the Mono.mac dataset using Slingshot. This approach infers a pseudotemporal ordering of cells, allowing us to model how cell types mature or transition over time. Three trajectories were predicted using the Slingshot algorithm, all originating from the Prolif.monocytes cluster (Figure 4E, Supplemental Figure 4B). Trajectory analysis revealed three distinct developmental pathways: one terminating in the Kupffer cell cluster (top right panel), a second terminating in the inflammatory macrophage (Inflam.mac) cluster (bottom right panel), and a third looping back to the proliferating monocyte cluster (Supplemental Figure 4C, right panel). The separation of Kupffer cells and inflammatory macrophages into distinct trajectories suggests that the expansion of the Kupffer cell cluster during infection is consistent with differentiation from circulating monocyte precursors, rather than reflecting a direct transition from inflammatory macrophages into the resident Kupffer cell niche, in line with previous descriptions of monocyte-derived Kupffer cell replenishment in the liver.”

3. Rationale for focusing on the Mono.mac cluster

The manuscript notes that Mono.mac is “one of three” clusters that express substantial levels of type I IFNs. However, the subsequent analysis (line 365) emphasizes Mono.mac specifically, without explaining why it is highlighted over the other two clusters. Clarification of the rationale for focusing on this cluster is needed.

We have added text to the end of line 311-313 to better explain the rationale behind beginning with the Mono.mac cluster analysis as follows:

“In contrast, hepatocytes from Ifnar1-/- Ifnlr1-/- mice failed to upregulate ISGs to the same extent, indicating that the antiviral response in this population is entirely dependent on IFN signaling. Together, these analyses identified four major clusters (Mono.mac, DC, hepatocytes, and neutrophils) that upregulated type I IFNs in response to echovirus infection across the four genotypes. They also demonstrate that type I IFNs are essential for driving a protective inflammatory response, particularly in Ifnar1-/- models. Given the cellular heterogeneity within these broad clusters, we next sought to resolve which specific subpopulations are responsible for IFN production across these four major clusters. Because the Mono.mac cluster harbored the highest number of viral reads in immunocompetent genotypes, we begin our subcluster analysis there, before moving on to the dendritic cell, hepatocyte, and neutrophil clusters.”

Reviewer #3: Line 326. Figure 3. The legend indicates L-M. However, Fig. 3 has A-N panels. This should read L-N.

Thank you, we will update this in the new version of the manuscript
